# Nitrogen Fertilizer Management for Enhancing Crop Productivity and Nitrogen Use Efficiency in a Rice-Oilseed Rape Rotation System in China

**DOI:** 10.3389/fpls.2016.01496

**Published:** 2016-09-30

**Authors:** Muhammad Yousaf, Xiaokun Li, Zhi Zhang, Tao Ren, Rihuan Cong, Syed Tahir Ata-Ul-Karim, Shah Fahad, Adnan N. Shah, Jianwei Lu

**Affiliations:** ^1^Key Laboratory of Arable Land Conservation (Middle and Lower Reaches of Yangtze River), Ministry of Agriculture, College of Resources and Environment, Huazhong Agricultural UniversityWuhan, China; ^2^Department of Crop Cultivation and Crop Physiology, College of Plant Science and Technology, Huazhong Agricultural UniversityWuhan, China

**Keywords:** nitrogen management, grain yield, rice-oilseed rape rotation, nitrogen use efficiency, apparent N balance

## Abstract

The use of efficient rates of nitrogen (N) fertilizer application is important with regard to increasing crop productivity and maintaining environmental sustainability. Rice-oilseed rape rotations are a mainstay of the economy and food security of China. Therefore, a field experiment was carried out during 2011–2013 in Honghu to identify the most appropriate N application rates for enhancing crop productivity and N use efficiency for rice (*Oryza sativa* L.)-oilseed rape (*Brassica napus* L.) rotations. Six N fertilizer treatments (RO1, RO2, RO3, RO4, RO5, and RO6) were laid out in a randomized complete block design with three replicates. RO_x_ represented the N fertilizer application rates (kg ha^−1^) for rice and oilseed rape, respectively. Grain yields from plots receiving N fertilizer were significantly increased by 59–71% (rice) and 109–160% (oilseed rape) during the total rotation (2011–2013), as compared to RO1 (control; no application). Furthermore, a similar trend was observed for N accumulation, ranging from 88 to 125% and 134 to 200% in aerial parts of rice and oilseed rape, respectively. Nitrogen use efficiency (NUE) was significantly higher (38.5%) under RO2 and lower (34.2%) under RO6 while apparent N balance (ANB) was positively lowest under R05 (183.4 kg ha^−1^) followed by R02 (234.2 kg ha^−1^) and highest under R06 (344.5 kg ha^−1^) during the total rotation. The results of grain yield, NUE, and ANB indicated that the R02 rate of N application was superior. This information should help to develop a cost-effective and environment-friendly N management strategy for rice-oilseed rape rotation systems of central China.

## Introduction

Arable farming has historically been dominated by attempts to achieve higher levels of production; however, new and diverse objectives now need to be considered. The environmental impact of crops and production systems, the quality of crop products, reduced costs of production and improved nitrogen (N) use efficiency are among the main objectives of modern agriculture.

Rice-upland rotations are important in south Asian countries (Yadav et al., 2000), and cover an estimated area of 26.7 million ha (Timsina and Connor, 2001). In China, these rotations contribute 72% of total cereal production and occupy an area of about 13 million ha. Globally, rice (*Oryza sativa* L.) is important for food security. In China, rice accounts for ~28% of the total grain-sown area and 43% of total grain production (Huang et al., 2002). With a constantly increasing population, it is estimated that Asian irrigated rice production needs to increase by 43% over the next 30 years (Cassman, 1999). However, a further expansion of rice hectarage will be difficult as most arable land is either already under rice production or has been converted into urban infrastructure (Horie et al., 2005). It is therefore necessary to constantly improve grain yield per unit area to maintain food security (Peng et al., 2006). Oilseed rape (*Brassica napus* L.) is globally the second most significant source of edible oil; it has high nutritional value and a favorable composition of fatty acids for both human consumption and livestock feed (Foley et al., 2011; Yousaf et al., in press). Therefore, its demand is increasing considerably all over the world (Kim et al., 2013). China is the world's leading producer of oilseed rape, which occupies 23.3% of the cultivated area and contributes 22.2% of global rapeseed production (FAO, 2010); it is therefore of great significance to the economy and food security of China.

The Yangtze River basin in China is a major zone for rice-oilseed rape rotation systems, contributing 4.3% (in 2010) of the total grain yield (Wang et al., 2012) and 91% of the national rapeseed (National Bureau of Statistics of China, 2012) production. In this area, oilseed rape is usually cultivated under either the single rice-oilseed rape system or the double rice-oilseed rape system, both of which limits the crop duration and soil nutrient supply accessible for increasing yield (Zhang et al., 2006). Furthermore, the yield of rice-upland rotations faces significant decline or stagnation as their sustainability is threatened by lower N use efficiency (Tian et al., 2013). Rice yield/ha in China is currently 50% higher than the global mean yield (FAO, 2010), whereas N fertilizer usage for rice (~190 kg ha^−1^) is 90% greater than global levels (Heffer, 2009). Because of the difficulty in predicting N fertilizer requirements, farmers often apply higher levels than those needed to maintain yield (Yadav et al., 2000; Ata-Ul-Karim et al., 2014a, 2016). However, excess N fertilizer is unlikely to be effective in increasing crop yields because of its diminishing returns (Tilman et al., 2011). Furthermore, this practice decreases N use efficiency (Peng et al., 2006; Ata-Ul-Karim et al., 2013, 2014b), causing a series of economic and environmental problems, as only an estimated 30–50% of applied N fertilizer is utilized by crops (Smil, 1999). N losses associated with higher application rates can result in leaching (Gheysari et al., 2009) that leads to contamination of surface and subsurface water (Barton and Colmer, 2006) and aquatic ecosystems (Fischer et al., 2010), emissions of N_2_O to the atmosphere (Huang and Tang, 2010) where it is a potent greenhouse gas (Stehfest and Bouwman, 2006), significant acidification in major croplands (Guo et al., 2010), and adverse effects on human health (van Egmond et al., 2002; Wilkinson et al., 2007). Therefore, the application of appropriate levels of N fertilizer through improved management is key to increasing N use efficiency (Tilman et al., 2002; Yousaf et al., 2014).

The effect of nitrogen on crops is profound; however, most understanding on crop growth responses to this element is empirical. An appropriate amount of N fertilizer encourages photosynthesis in both oilseed rape (Hu et al., 2007) and rice plants (Hussain et al., 2016), it enhances resistance to biotic stress (Guo et al., 2009), improves dry matter accumulation and nutrient uptake (Barłóg and Grzebisz, 2004a,b), and increases grain and oil yields (Juan et al., 2009); meanwhile, over-fertilization has become a major concern for sustainable intensive agriculture in China (Meng et al., 2013).

To-date, few studies have focused on N fertilizer management and the efficiency of its application to rice and oilseed rape crops. Furthermore, to the best of our knowledge, no one has reported on these factors in rice-oilseed rape rotations, especially in China. Therefore, the present study was conducted to develop an improved N management strategy for rice-oilseed rape systems in central China, to quantify appropriate N fertilizer rates for enhancing crop productivity, and to evaluate the contribution of applied N to N use efficiency and apparent N balance. The results will be useful in developing cost-effective and environmentally-friendly N management strategies for the rice-oilseed rape systems of central China.

## Materials and methods

### Description of experimental area

**Figure 1:**
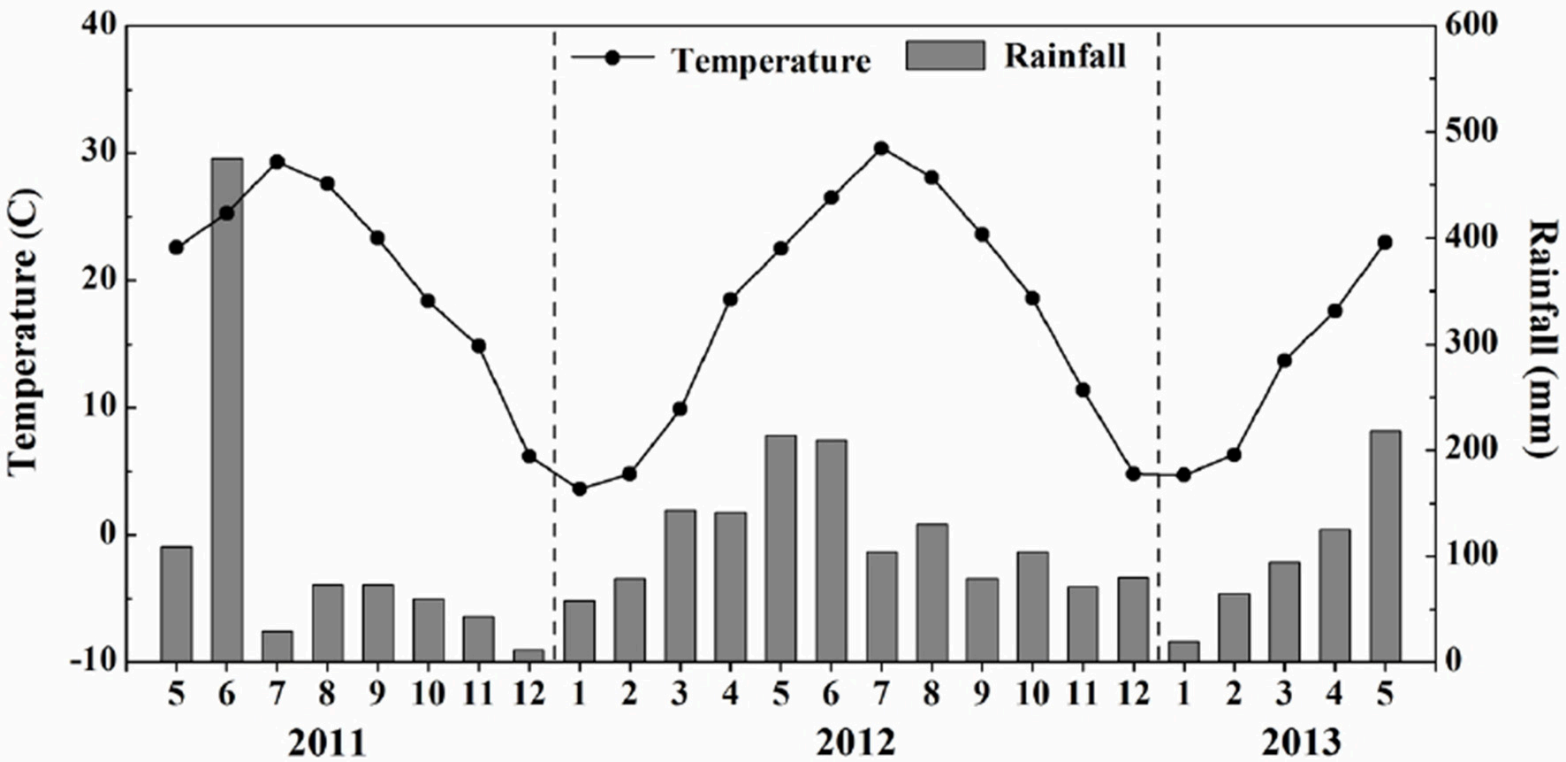
**Monthly total rainfall and monthly mean temperature during the crop growing season in the experiment conducted during 2011–2013**.

The two-year field experiment was conducted during 2011–2013 on a rice and winter oilseed rape rotation at Honghu in the Hubei province of China (30°01′N, 113°32′E). The region has a subtropical climate with mean temperatures ranging from 6.2 to 29.3°C in 2011, 3.6 to 30.4°C in 2012, and 4.7 to 23.0°C in 2013. In the same years, rainfall varied from 11.7 to 475.3 mm, 57.9 to 213.8 mm, and 19.2 to 217.8 mm, respectively. During the winter oilseed rape growing season, the temperature was mostly low (4°C or lower) with little precipitation (< 100 mm) between January to February (Figure 1).

### Experimental design and operation

The experiment was laid out in a randomized complete block design in three replicates with six treatments as follows: No nitrogen applied during either season of rice or oilseed rape (R01); 180 kg ha^−1^ N applied during each season of rice and oilseed rape (R02); 210 kg ha^−1^ N for the rice season and 150 kg ha^−1^ N for the oilseed rape season (R03); 150 kg ha^−1^ N for the rice season and 210 kg ha^−1^ N for the oilseed rape season (R04); 150 kg ha^−1^ N (R05) and 210 kg ha^−1^ N (R06) applied during each season of rice and oilseed rape. All six treatments received calcium superphosphate (P 5.2%) and potassium chloride (K 52.3%) at the uniform rate of 60 and 90 kg ha^−1^, respectively. N fertilizer in the form of urea (N 46.4%) was applied to rice in three splits: 50% as a base fertilizer, 25% at tillering stage, and 25% at panicle initiation stage. For oilseed rape seasons, 60% was applied as a base fertilizer, 20% in the over-wintering stage, and 20% at the initiation of stem elongation (Wang et al., 2014). All P fertilizer was applied as a base in each cropping season of both crops. K fertilizer was applied as follows: 70% before the sowing of rice and oilseed rape, 30% at panicle initiation, and 30% at the top dressing stage for rice and oilseed rape seasons, respectively. Borax was added at a rate of 15 kg B ha^−1^ as a basal application for the oilseed rape season only, to meet the nutrient requirements for normal growth (Wang et al., 2014). Replicate plot sizes measured 20 m^2^ (3 m × 6.7 m) for both crops.

The experimental field was well prepared, plowed and leveled with a rotary plow, and basal fertilizers were incorporated during the final plowing. Straw residues of previous crops were completely removed from the fields before the sowing of each crop. Local varieties, Y-liangyou1 (rice), and Hua youza9 (oilseed rape) were chosen as experimental crops; they are widely cultivated in the locality due to high-yields and extensive adaptability. The nursery was raised near the experimental site on a fertile seedbed and was transplanted to the field after 30 days of seedling emergence for each growing season. All other field operations such as planting density, irrigation, herbicide application, and disease and pest control were performed using local methods. No major attack of weeds, disease, pest, or inclement weather was recorded during the growing seasons. Plant densities were kept uniform at 200,000 and 112,500 ha^−1^ for both growing seasons of rice and oilseed rape, respectively. Seeding, transplanting, and harvest times are shown in Table 1.

**Table 1:** **Timing of each operation for rice and oilseed rape in the experiment conducted during 2011–2013**.

**Operation**	**1st rotation (2011–2012)**		**2nd rotation (2012–2013)**	
	**Rice**	**Oilseed rape**	**Rice**	**Oilseed rape**
Seeding	8 May 2011	25 Sept. 2011	9 May 2012	20 Sept. 2012
Transplanting	11 Jun. 2011	25 Oct. 2011	12 June 2012	21 Oct. 2012
Harvesting	20 Sept. 2011	15 May 2012	22 Sept. 2012	13 May 2013

### Sampling and measurement

Soil samples were collected at depths of 0–20 cm at 20 random points before the commencement of experiment to analyze the physical-chemical properties of study site. A sub-sample of fresh soil was used for the measurement of inorganic N (Rowell, 1994). The remaining soil was air-dried and ground to pass through a 2-mm sieve for measurement of pH (1:2.5 soil/water ratio), organic C (dichromate oxidation method), total N (Kjeldahl acid-digestion method), Olsen-P by spectrophotometer, NH_4_OAc-K by flame photometer, and soil type using the hydrometer method. The experimental soil was silty clay loam in texture with a pH of 7.47. Organic matter and total nitrogen was 24.2 g kg^−1^ and 1.93 g kg^−1^, respectively, while Olsen-P was 6.9 mg kg^−1^, and NH_4_OA_C_-K was 96.1 mg kg^−1^.

To investigate the overall effects of fertilizer application, plants were sampled at maturity of both crops to determine plant dry matter (kg ha^−1^) and nutrient uptake (kg ha^−1^; Wang et al., 2014). Plant samples were washed with deionized water and divided into seeds, stems, pod walls for oilseed rape and grains, and straw for rice. Each aerial fraction was separately chopped and dried to a constant weight at 65°C, then all dried and milled plant samples were digested with H_2_SO_4_–H_2_O_2_. Total plant N concentrations (%) were determined using an automated continuous flow analyzer (AA3, Bran and Luebbe, Nordersted, Germany). N uptake was calculated by multiplying the crop dry matter by N concentrations in aerial plant parts. At maturity, rice and rapeseed were harvested manually from each plot and yields were adjusted to a moisture content of 14 and 8–12%, respectively.

### Data analysis

Analysis of variance (ANOVA) was conducted on data separated for each year. The data were statistically analyzed using SPSS 17.0 (IBM) software. Differences between the treatments were calculated using the least significance difference test (LSD) at 0.05 probability level. Figures were prepared using MS Excel (Microsoft Office 2007) and Origin 8.0 (Origin Lab) software.

### Calculation methods

#### Apparent nitrogen balance (ANB)

Mean annual apparent balance values for plant N was calculated using the method of Duan et al. (2014).

$$\begin{array}{l} {\text{ANB kg ha}}^{-1}=\text{Total N uptake}-\text{N applied} \end{array}$$

#### Nitrogen use efficiency (NUE)

NUE was calculated according to Su et al. (2014).

$$NUE=\frac{Difference\;in\;N\;uptake\;with\;and\;without\;N\;applied}{N\;application\;rate}$$

## Results

### Yield of rice and oilseed rape in response to nitrogen fertilization

Results indicated a significant effect of different N fertilization rates on rice and oilseed rape yield (Table 2). In 2011–2012, yields varied from 5302 to 8955 kg ha^−1^ and 550 to 1384 kg ha^−1^, respectively, while in 2012–2013, they were 4781 to 8424 kg ha^−1^ and 793 to 2145 kg ha^−1^, respectively. Rotation yields were statistically similar and higher under the R06 and R02 treatments while the lowest yields were observed under the R01 treatment (see Table 2). The greatest yield increase for rice was obtained at a rate of 210 N kg ha^−1^; however, this was not significantly different from the application of 180 N kg ha^−1^. The yield-increase ratio for oilseed rape was highest at a rate of 180 N kg ha^−1^. In contrast, the lowest yield increase in the rice-oilseed rape rotation was observed in the R05 treatment that received the lowest application rate. With the different rates of N application, overall, the total rotation yield (2011–2013) varied from 10,083 to 17,229 kg ha^−1^ and 1344 to 3496 kg ha^−1^ for rice and rapeseed, respectively. When compared to the R01 treatment, other treatments increased total yields by 59 to 71% and 109 to 160% for rice and rapeseed, respectively. These results indicated that a balanced N application of 180 kg ha^−1^(R02) was superior and increased the total yield by 69% for rice and 156% for rapeseed when compared to the control (R01).

**Table 2:** **Grain and seed yields (kg ha^−1^) of rice and oilseed rape as affected by different application rates of nitrogen (N) fertilizer in the experiment conducted during 2011-2013**.

**Treatment**	**1st rotation (2011-2012) kg ha^−1^**				**2nd rotation (2012-2013) kg ha^−1^**				**Total rotation (2011-2013) kg ha^−1^**			
	**Rice**	**Increment to R01 (%)**	**Oilseed rape**	**Increment to R01 (%)**	**Rice**	**Increment to R01 (%)**	**Oilseed rape**	**Increment to R01 (%)**	**Rice**	**Increment to R01 (%)**	**Oilseed rape**	**Increment to R01 (%)**
R01	5302c		550d		4781e		793c		10083c		1344c	
R02	8603ab	62	1384a	152	8424a	76	2060a	160	17028a	69	3444a	156
R03	8955a	69	1201b	118	8146bc	70	1777b	124	17102a	70	2977b	122
R04	8413b	59	1351a	146	7987c	67	2145a	171	16400b	63	3496a	160
R05	8325b	57	1076c	96	7751d	62	1735b	119	16076b	59	2811b	109
R06	8921a	68	1376a	150	8307ab	74	1988a	151	17229a	71	3364a	150

*Mean values within a column for each season followed by different letters are significantly different at P < 0.05 according to LSD*.

### Nitrogen uptake in response to N fertilization and N contribution to rice and rapeseed crop

Total N uptake in the aerial parts of rice and oilseed rape under different N fertilization levels are shown in Table 3. Total N uptake was significantly enhanced under different levels of N fertilization compared with the R01 treatment. In 2011–2012, total N varied from 82 to 202 kg ha^−1^ (rice) and 20 to 64 kg ha^−1^ (oilseed rape), while in 2012–2013, the total N uptake by aerial plant parts ranged between 71 to 155 kg ha^−1^ and 34 to 105 kg ha^−1^, respectively. Highest N uptake was observed under the R06 followed by the R03 and R02 treatments during the 2011–2012 rotation. During the 2012–2013 rotation, N uptake was highest under R02 followed by the R06 and R04 treatments. The lowest N uptake in all parts of both crops during both rotation years was recorded under R01. When compared to the R01 treatment, other application rates showed increased total N uptake from 2011 to 2013 by 88 to 125% (rice) and 134 to 200% (oilseed rape). Furthermore, the increment ratio of N uptake by rice and oilseed rape was highest under R02 in the 2nd, and total rotation; in the 1st rotation, the N uptake increment ratio was greatest under the R06 followed by R02 treatments. Hence, results illustrated that N accumulation at the R02 level was the optimal rate.

**Table 3:** **Nitrogen (N) uptake (kg ha^−1^) transmitted to aerial parts of rice and oilseed rape plants as affected by different rates of N fertilizer application in the experiment conducted during 2011–2013**.

**Treatment**	**1st rotation (2011–2012) kg ha^−1^**				**2nd rotation (2012–2013) kg ha^−1^**				**Total rotation (2011–2013) kg ha^−1^**			
	**Rice**	**Increment to R01 (%)**	**Oilseed rape**	**Increment to R01 (%)**	**Rice**	**Increment to R01 (%)**	**Oilseed rape**	**Increment to R01 (%)**	**Rice**	**Increment to R01 (%)**	**Oilseed rape**	**Increment to R01 (%)**
R01	82e		20c		71c		34d		154c		55d	
R02	174bc	111	64a	216	155a	117	94b	173	328a	114	157ab	189
R03	190ab	131	51b	154	143ab	100	78c	128	333a	117	129c	137
R04	162cd	96	59a	193	131b	84	105a	204	293b	90	164a	200
R05	151d	83	49b	145	139b	94	78c	127	289b	88	127c	134
R06	202a	145	59a	196	144ab	101	91b	164	345a	125	150b	175

*Mean values within a column for each season followed by different letters are significantly different at P < 0.05 according to LSD*.

The distribution of N to aerial parts of rice and oilseed rape in response to N fertilizer levels was calculated (see Table 3). In 2011–2012, ~73 to 80% of the N absorbed was allocated to aerial parts of rice, and the remaining 20 to 27% of absorbed N was utilized by the aerial biomass of oilseed rape; while in 2012–2013, the ratio was 56 to 67% and 33 to 44%, respectively. Compared with oilseed rape, the higher allocation of N fertilizer to rice indicated that rice requires more nitrogen than the former; however, the oilseed rape showed greater sensitivity to N fertilizer; this was also reflected by the increment ratio of yield and N uptake between rice and oilseed rape (Tables 2, 3).

### Nitrogen use efficiency and apparent N balances in response to N fertilization

**Figure 2:**
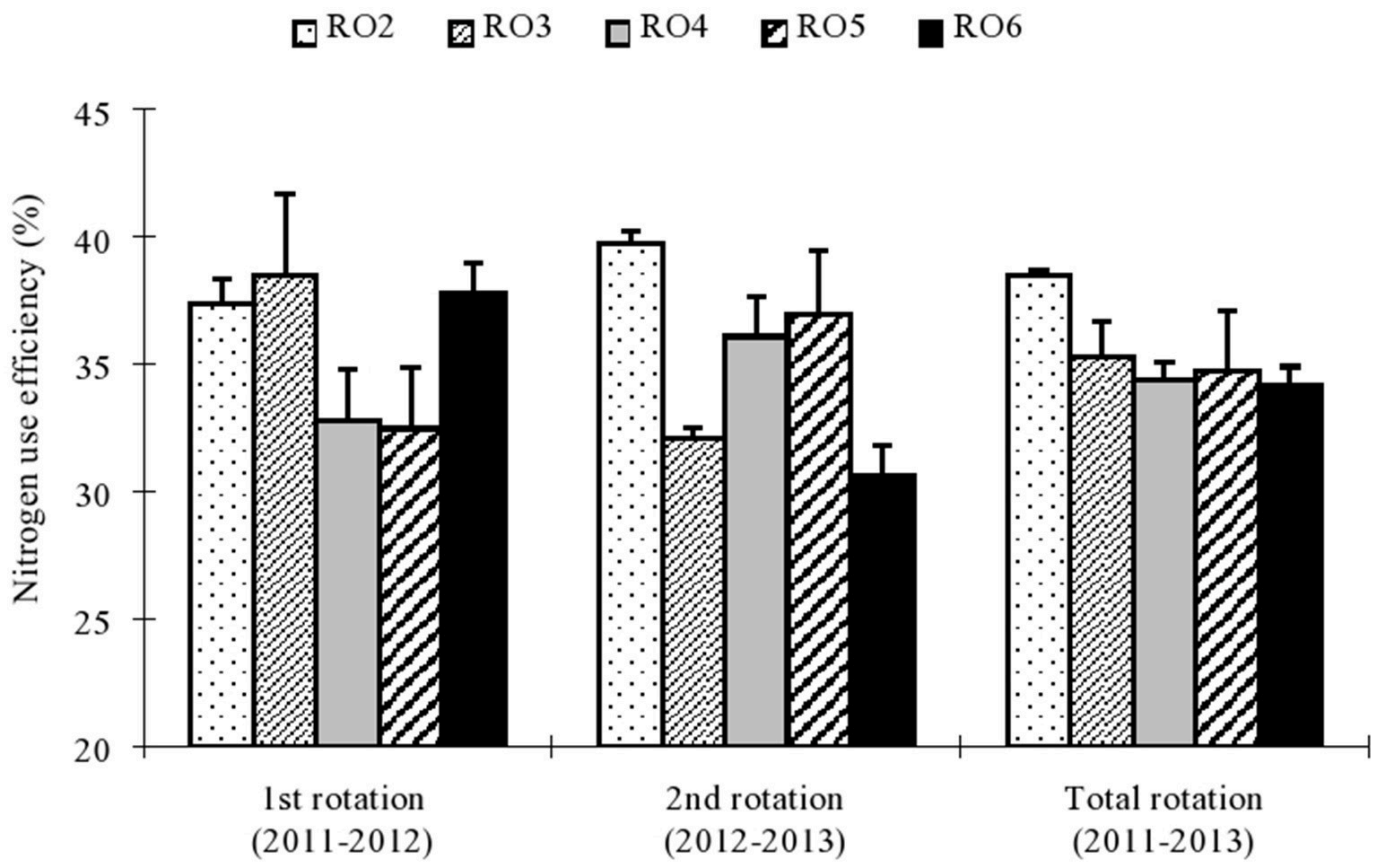
**Nitrogen (N) use efficiency (NUE %) of rice and oilseed rape as affected by different N application rates in the 1st (2011–2012), 2nd (2012–2013), and total rotation (2011–2013)**. Each value represents the standard error (*n* = 3). Within a season, bars with different letters are significantly different at *P* < 0.05 according to LSD.

Nitrogen use efficiency was significantly influenced by different application rates during the rotations (Figure 2). During 2011–2012, NUE ranged from 32 to 38% while in 2012–2013, it ranged from 31 to 40%. Maximum total NUE during 2011–2013 was observed for R02 (39%) while the lowest was observed under R06 (34%). These results showed that N fertilization under R02 improved NUE, and reduced N loss and environmental risk.

**Figure 3:**
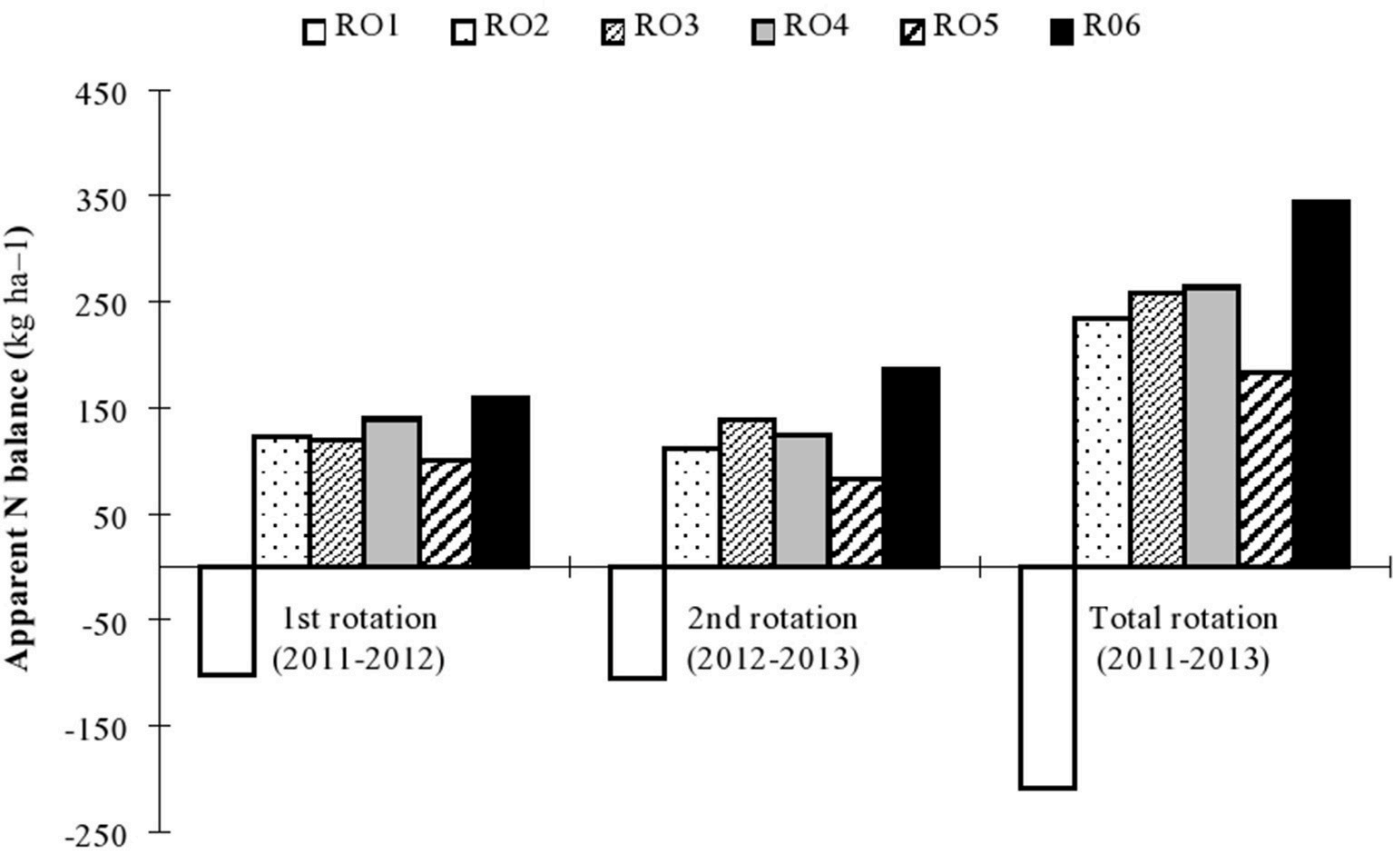
**Apparent nitrogen (N) balance (kg ha^−1^) of rice and oilseed rape as affected by different N application rates in the 1st (2011–2012), 2nd (2012–2013), and total rotation (2011–2013) in the experiment conducted during 2011–2013**.

Apparent N balance values also varied for different N fertilizer rates ranging from −102.5 to 159.0 kg ha^−1^ during 2011–2012 and from 105.9 to 185.5 kg ha^−1^ during 2012–2013 (Figure 3). Furthermore, N balance was affected by application rates as follows: R06>R04>R02>R03>R05>R01, during 2011–2012, and R06>R03>R04>R02>R05>R01 in 2012–2013. ANB during the overall rotation was highest under R06 (344.5 kg ha^−1^) while lowest under R05 (183.4 kg ha^−1^) followed by R02 (234.2 kg ha^−1^). Hence, application rates that lowered N threat to the environment could be ranked as R05>R02>R06; the lowest was under R01 that had no N input. Negative ANB values were only observed for the R01 treatment.

## Discussion

### Rice and oilseed rape yield

Efficient nitrogen fertilizer management is essential for achieving economic yields and for enhancing N use efficiency (Pan et al., 2012). Normally, N fertilization will raise grain yield and increase growers' profits. However, high application rates are not guaranteed to continually increase yield, and might result in low N use efficiency or environmental issues (Guo et al., 2010; Tilman et al., 2011).

Sustainable crop production relies on the continuous renewal of soil fertility through a balance between N demand and supply in cropping systems. N is the most yield-restraining nutrient in crop production globally (Guo et al., 2016); it is required in the greatest quantity by plants and is the most mobile element in the soil (Timsina et al., 2006). However, N availability, accumulation, and its utilization by crops are restricted by various biotic and abiotic components in the soil-plant system such as fertilizer application rates, cultivar, climate, irrigation, crop residue, and crop management (Witt et al., 2000; Yadvinder-Singh et al., 2005). The current results showed variable grain yields in both crops under different N application rates during both rotation years; this illustrates the significance of N for boosting crop productivity and agrees with previous reports on rice and oilseed rape (Wang et al., 2012; Li et al., 2015). Differences in yield were mainly the consequent of different N rates associated with soil fertility and the N uptake ratio by aerial parts of rice and oilseed rape (Table 3). These results are in agreement with previous studies reported by Zhang et al. (2003) and Pan et al. (2012), who reported that yield components were affected by the rate of N fertilization, and that crop yields are usually more dependent on fertilizer levels (Harrison and Webb, 2001) and soil fertility (Hossain et al., 2005). A close correlation between N uptake and crop yield has also been documented by Witt et al. (2000) and Timsina et al. (2006). The current results illustrated that N released under the R02 treatment met the requirements of rice and oilseed rape, which in turn resulted in the efficient translocation of photosynthetic products during grain formation and consequently led to an increase in yield.

### Nitrogen use efficiency and N uptake

Synchronization of crop N requirements with N supply is key to improving N use efficiency. A crop's demand for N is firmly related to yield potential, which in turn is associated with N supply and crop management practices. In this study, different N application rates significantly increased NUE and in particular, the R02 treatment was superior when compared with other application rates in the 1st, 2nd, and total rotation of rice and oilseed rape (Figure 2). Rice NUE was high (46 to 57%) in the 1st rotation and low (34 to 46%) in 2nd rotation; while for oilseed rape, NUE was low (18 to 24%) in 1st rotation and high (27 to 33%) in 2nd rotation. This was assumed to be due to greater N accumulation in rice as compared to oilseed rape, which was also reflected in the allocation of N uptake by aerial parts of the crops where 64 to 80% of absorbed N was used by rice, and the remaining 20 to 44% was utilized by oilseed rape. The ratio of N accumulation by aerial parts of rice was greater in the 1st rotation, while that of oilseed rape was lower in the 1st rotation (Table 3). N application raises N uptake, and application rates of 240 N kg ha^−1^ enhanced N uptake compared with the 150 N kg ha^−1^ in rice plants (Pan et al., 2012); similarly, it has been shown that any preceding crop also influences N accumulation in canola (Luce et al., 2016). Higher N use efficiencies were possibly obtained in the R02 treatment because the level of applied N closely matched the N requirements of rice and oilseed crops; this level of application achieved an increase in N uptake and crop yields while reducing N losses to the environment. Dawe et al. (2003) found that N losses decrease with improved yield and NUE in rice, and similar results are reported by Fofana et al. (2005) in maize crops. Moreover, a greater panicle N fraction significantly enhances NUE, regardless of cultivar or growing season (Jiang et al., 2004). Elements influencing crop N use efficiency include crop, accessibility of other nutrients, nutrient leaching, weather, and genotypic differences (De-shui et al., 2007; Takahashi and Anwar, 2007). All of these components may also responded to the differences in NUE of rice and oilseed rape in different rotations.

### Fate of applied nitrogen

Better management of high yielding crops with lower N loss is desperately needed to achieve sustainable Chinese agriculture (Duan et al., 2014). In the current study, the fate of N fertilizer was evaluated using ANB. During both rotation seasons of rice-oilseed rape, ANB response to applied N fertilization levels was 83–186 kg N ha^−1^, which is comparable to the 24–190 kg N ha^−1^ in a rice-wheat system in Bangladesh (Timsina et al., 2006). It has been established that N losses increase as N fertilizer input increases up to 200 kg N ha^−1^ (Sepaskhah and Tafteh, 2012). The results of the current study showed that the R02 application rate, with an ANB value of 234 kg N ha^−1^ during the total rotation, was sufficient to sustain rice and oilseed rape yields. The R02 application rate improved NUE and N accumulation as compared to the ANB value of 345 kg N ha^−1^ under the R06 treatment. This management change would result in saving at least 16% yr^−1^ of N resources with significantly lower N losses to the environment in the rice-oilseed rape rotation. The most important N losses are ammonia volatilization during rice-growing seasons (Xu et al., 2013; Duan et al., 2014) while N leaching and denitrification are the main losses during oilseed rape-growing seasons (Sepaskhah and Tafteh, 2012; Luce et al., 2016). The current results agree with the reports of various researchers who observe that ANB and N losses respond to N fertilization during crop rotations (Timsina et al., 2006; Duan et al., 2014; Luce et al., 2016).

## Conclusion

Economic management of N fertilizer application is essential for improving crop productivity, N use efficiency, and environmental sustainability. In the current study, rice and oilseed rape yields were significantly higher in N fertilized plots. Higher positive ANB values demand the application of reasonable and efficient N fertilizer inputs to avoid loss to the environment and to improve NUE and N uptake for better rice-oilseed rape productivity. Results indicated that a combination of external N input and crop N uptake is key to maintaining productivity in rice-oilseed rape systems. Furthermore, based on yields, NUE, and ANB, the R02 application rate was superior for rice-oilseed rape rotations in the study region. Further studies are required on N fertilizer distribution by using R_180_–O_150_ and R_150_–O_180_ to address the variability of economic income and the risk of environmental pollution. The findings of the current study can be used to develop appropriate N management strategies for rice-oilseed rape rotations in central China. Additional studies under various N management practices using different cultivars in different rice-oilseed rape rotation systems would be supportive.

## Author contributions

XL, TR, and RC initiated and designed the research, MY, ZZ, and AS performed the experiments and collected the data, MY, XL, and JL analyzed the data and wrote the manuscript. JL, SA, and SF edited the manuscript and provided guidance during experimentation.

## Funding

This research was supported by National Natural Science Foundation of China (41401324), Special Fund for Agro-scientific Research in the Public Interest (201303103), and the Fundamental Research Funds for the Central Universities (2662015PY135).

### Conflict of interest statement

The authors declare that the research was conducted in the absence of any commercial or financial relationships that could be construed as a potential conflict of interest.
